# Are PD-1 inhibitors effective for recurrent/metastatic nasopharyngeal carcinoma? Meta-analysis and systematic review

**DOI:** 10.3389/fphar.2022.1095734

**Published:** 2023-01-09

**Authors:** Le Yan, Bi Ren, Rongqiu Hu, Huiping Zhang, Haocheng Gou

**Affiliations:** ^1^ School of Medical and Life Sciences, Reproductive and Women-Children Hospital, Chengdu University of Traditional Chinese Medicine, Chengdu, China; ^2^ North Sichuan Medical College, Affiliated Hospital of North Sichuan Medical College, Nanchong, China; ^3^ School of Nursing, Southwest Medical University, Luzhou, China; ^4^ Department of Otorhinolaryngology Head and Neck Surgery, The Second Clinical Medical College, Nanchong Central Hospital, Nanchong, China

**Keywords:** recurrent/metastatic nasopharyngeal carcinoma, PD-1, meta-analysis, adverse event, ICB

## Abstract

**Objective:** For metastatic/recurrent nasopharyngeal carcinoma (NPC) patients, a programmed cell death protein 1 (PD-1) is a controversial option. This meta-analysis aimed to investigate the efficacy and safety of PD-1 inhibitors in patients with metastatic/recurrent NPC.

**Methods:** Electronic databases such as PubMed, Embase, Cochrane library, and Web of Science were manually searched until 1 July 2022, and Stata 15.0 was used to analyze the data.

**Result:** A total of 10 studies were included, of which three were randomized controlled trials with data, and seven were single-arm studies. For randomized controlled trial (RCT) study, ORR [OR = 1.11, 95% CI (.49, 2.52); *p* = .812], OS [1-year OR = 1.26, 95% CI (.76, 2.08); *p* = .367], [2-year OR = 1.04, 95% CI (.39, 2.71); *p* = .928] in patients with metastatic/recurrent NPC were consistent with PD-1 inhibitor therapy and conventional chemotherapy. However, PD-1 inhibitor had higher 1-year PFS than conventional chemotherapy [OR = 2.16, 95% CI (1.26, 3.70); *p* = .005]. For single-arm studies, after PD-1 inhibitor therapy, the ORR of patients with recurrent/metastatic NPC reached [ES = 37%, 95 CI (17%–56%)], 1-year OS [ES = 61%, 95% CI (46%–76%)], 2-year [ES = 16%, 95% CI (6%–26%)], and 1-year PFS [ES = 16%,95% CI (12%–20%)].

**Conclusion:** The efficacy of PD-1 inhibitor monotherapy in patients with metastatic/recurrent nasopharyngeal carcinoma was not significantly different from that of conventional chemotherapy; however, due to the limitations of the included studies, further phase III RCTs are required to corroborate our conclusion.

**Systematic Review Registration:** https://www.crd.york.ac.uk/prospero/display_record.php?ID=CRD42022342400; Identifier: CRD42022342400.

## 1 Introduction

Nasopharyngeal carcinoma (NPC) is a malignant tumor that originates from the nasopharyngeal mucosal epithelium (Chen et al., 2019). The histological types are mostly poorly differentiated or undifferentiated carcinomas. The incidence is high in southern China and North Africa (Beyene et al., 2021; Bossi et al., 2021). According to GLOBOCAN (Bray et al., 2018), the incidence in these regions is 4–25 cases per 1,00,000 people, which is 50–100 times higher than the incidence in the rest of the world. NPC can be divided into three subtypes: keratinizing squamous cell carcinoma, non-keratinizing squamous cell carcinoma, and undifferentiated or poorly differentiated carcinoma (Kang et al., 2020; Yarza et al., 2021). The non-keratinizing subtype of NPC accounts for 95% of NPC endemic areas and 75% in the USA. This unique geographic distribution has been linked to genetic and environmental factors (Chang et al., 2021). NPC is a high radio- and chemo-sensitive tumor type (Lee et al., 2015). NPC is sensitive to both chemoradiation and chemotherapy except in stage I patients (Kang et al., 2020; Tsang et al., 2020). Currently, gemcitabine and/or cisplatin are the standard (first-line) treatments for NPC (Zhang et al., 2019). The 5-year OS of patients with early-stage NPC after receiving standard chemotherapy regimens can be as high as 80% (Dwijayanti et al., 2020); for patients with recurrent or metastatic NPC, the 5-year OS is only 40%–50% (Lee et al., 2019; Sun et al., 2019). Therefore, patients with recurrent/metastatic nasopharyngeal carcinoma are less effective in first-line therapy, and second-and third-line treatment options are limited.

Tumor immunotherapy has the potential to activate the body’s immune system while also targeting cancer cells and tumor tissues, making it an essential option for tumor therapy (Riley et al., 2019; Hiam-Galvez et al., 2021). Clinically, immunotherapy can be divided into two categories: the first category refers to active immunotherapy methods, such as adoptive immunotherapy or tumor vaccines (Gohil et al., 2021); the second category refers to the host’s natural anti-tumor immune response (Li et al., 2019), immune suppression or escape mechanisms. Blocking the inhibitory pathway of infiltrating T cells reactivates antitumor immune response, known as an immune checkpoint blockade (ICB) (Diesendruck and Benhar, 2017). At present, immune checkpoint inhibitors mainly targeting PD-1 play a role in the process of ICB (Kumar et al., 2020). ICB has become a research hotspot, and the most well-known target molecule in ICB therapy is PD-1. There are many ongoing phase III trials (NCT03427827, NCT04376866, NCT04446663, NCT04447612) to compare the efficacy of PD-1 inhibitors and chemotherapy for metastatic/recurrent NPC, and clinical opinions on PD-1 inhibitors for metastatic/recurrent NPC are inconsistent. The objective of this meta-analysis was to analyze and summarize the currently available data on the efficacy and safety of PD-1 inhibitors alone or in combination with chemotherapy in the treatment of patients with metastatic/recurrent NPC to determine the efficacy of this class of drugs and provide new treatment options for patients and clinicians.

## 2 Materials and methods

The protocol has been registered in the International Prospective Register of Systematic Reviews database (PROSPERO: CRD42022342400).

### 2.1 Retrieval strategy

Search PubMed, Embase, Cochrane library, and Web of science for articles published by 1 July 2022, on PD-1 inhibitors for metastatic/recurrent NPC. The search terms are (Nasopharyngeal Carcinoma, Carcinomas, Nasopharyngeal, nasopharyngeal cancer, NPC) and (Checkpoint Blockers, Immune, PD-1 Inhibitors), specific searches strategy of PubMed and Embase see Supplementary Tables S1, S2.

### 2.2 Inclusion and exclusion criteria

The included population was diagnosed with recurrent nasopharyngeal carcinoma or distant metastasis of nasopharyngeal carcinoma and received PD-1 inhibitor intervention. Randomized controlled studies (RCT) or single-arm studies reporting OS: overall survival, PFS: progression-free survival, ORR: objective response rate were included, and complete response (CR) + partial response (PR) are considered ORR. Conference abstracts, literature reviews, meta-analyses, duplicate publications, animal experiments, case reports, the number of included cases <10, the full text not available, and the data not available were all excluded.

### 2.3 Data extraction

The extracted data included the investigator’s name, publication year, drug type, number of included cases, drug dose, follow-up, median OS, median PFS, and median ORR. The basic information of the studies was extracted independently by two investigators.

### 2.4 Risk of bias evaluate

For RCTs: the Cochrane to Randomized Clinical Trials Risk of Bias Tool 2.0 (RoB2) was used to assess the risk of bias (Sterne et al., 2019). RoB2 was also paired with two independent investigators. A third investigator performed consensus if two investigators differed on the risk of bias analyzed. The evaluators examined the randomization process, deviations from expected interventions, missing outcome data, choice of outcome measures, and reported outcomes. Therefore, the studies were classified as low, moderate, or high risk of bias (Supplementary Figure S1).

For single arm study: The Newcastle-Ottawa Scale (NOS) (Lo et al., 2014) was used to assess quality. Assessment scores of 0–3, 4–6, and 7–9 represent poor, fair, and good studies, respectively, and any disagreements are resolved by consensus (Supplementary Table S3).

### 2.5 Data analysis

For randomized controlled trials, Odds ratio (OR) and 95% confidence interval (CI) were used for binary variables such as ORR value, 1-year OS, 2-year OS and 1-year PFS. For single-arm studies, we used effect size (ES) and 95% CI. The random utility model was employed for meta-analysis due to the considerable heterogeneity of treatment types, frequency, and frequency among different studies. Stata software (version 15.0; Stata Corp, College Station, TX, United States) performed statistical analyses and tested for heterogeneity by I2 values or Q statistics. I2 values of 0%, 25%, 50%, and 75% represent no, low, medium and high heterogeneity, respectively. Sensitivity analyses were undertaken when I2 values were ≥50% to investigate potential sources of heterogeneity; otherwise, the fixed effects model was employed. In addition, publication bias was assessed by Egger’s test or Begg’s test using the random effects model. Two-sided *p* < .05 was considered statistically significant.

## 3 Result

### 3.1 Literature screening and characteristics

**FIGURE 1:**
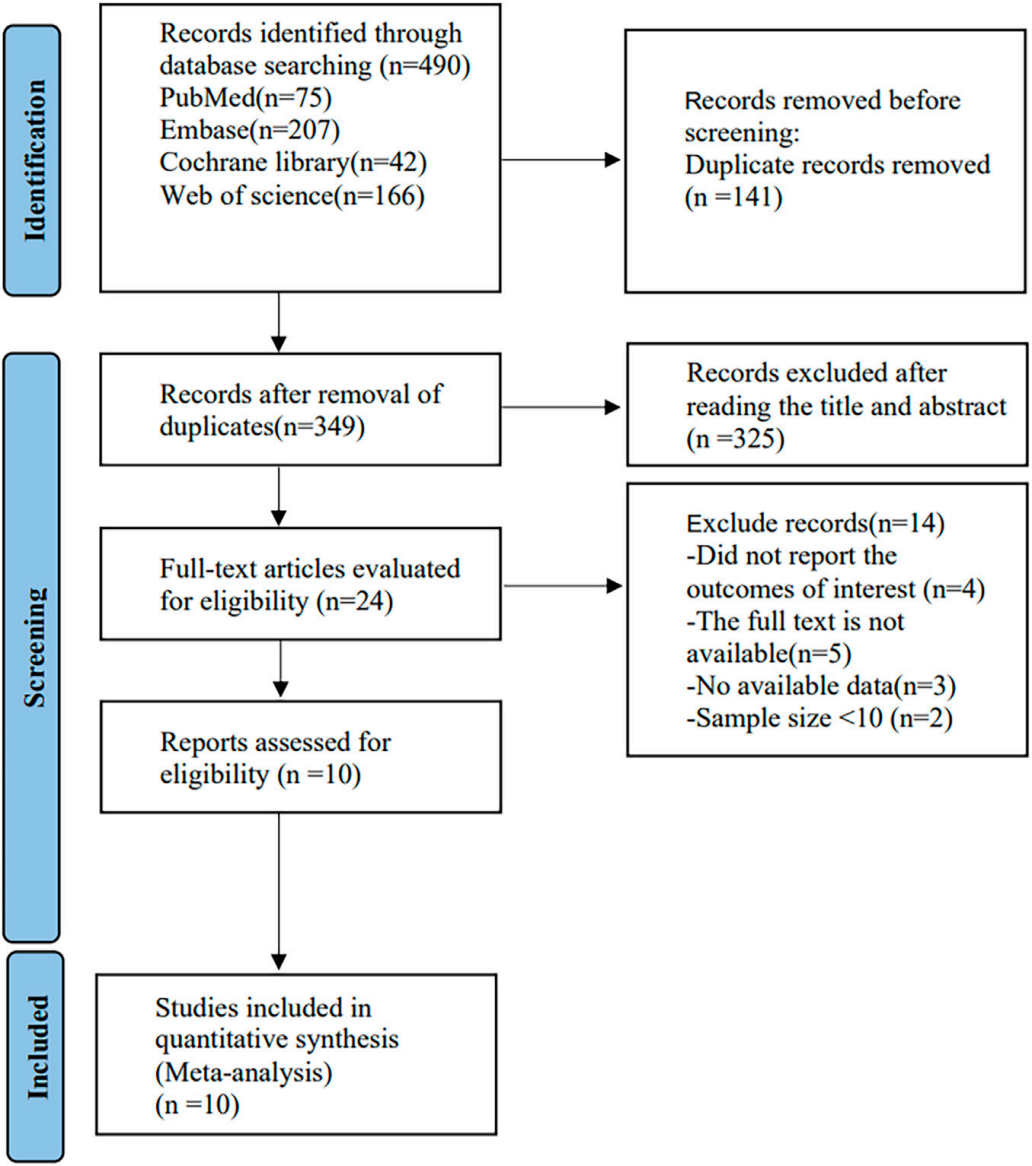
Literature screening flowchart.

Through manual retrieval, a total of 490 articles were obtained, 349 articles were obtained after removing duplicates, 24 articles were obtained by checking the titles and abstracts of the articles, 10 articles (Hsu et al., 2017; Fang et al., 2018; Ma et al., 2018; Sato et al., 2020; Even et al., 2021; Ma et al., 2021; Mai et al., 2021; Wang et al., 2021; Yang et al., 2021; Economopoulou et al., 2022) were finally included in the analysis by reading the full text. See Figure 1.

### 3.2 Characteristics of literature

A total of 10 studies were included, of which 3 (Even et al., 2021; Mai et al., 2021; Yang et al., 2021) were randomized controlled trials with data, and 7 (Hsu et al., 2017; Fang et al., 2018; Ma et al., 2018; Sato et al., 2020; Ma et al., 2021; Wang et al., 2021; Economopoulou et al., 2022) were single-arm studies. The most used PD-1 inhibitors included: Camrelizumab, Toripalimab, Pembrolizumab, and Nivolumab. There are four studies (Fang et al., 2018; Sato et al., 2020; Mai et al., 2021; Yang et al., 2021) that did not report the median OS, and the specific characteristics of the literature are shown in Table 1.

**TABLE 1:** Literature baseline table.

Study	Year	Type	Sample size (male)	Mean age (years)	Dose	Follow-up (Mo)	Median OS(Mo)	Median PFS(Mo)	Median ORR (%)	Metastatic sites	PD-L1>1%	TNM stage
Randomized controlled trial												
YP Yang	2021	PD-1	T:134 (113)	T:52	Camrelizumab 200 mg Q3W	20	NR	T:9.7	T:87.3	Liver; lung	NR	NR
			C:129 (105)	C:49				C:6.9	C:80.6			
HQ Mai	2021	PD-1	T:146 (124)	T:46	Toripalimab 240 mg Q3W	30	NRE	T:11.7	T:77.4	Liver; lung; bone	T:109	NR
			C:143 (116)	C:51				C:8	C:66.4		C:109	
Even C	2021	PD-1	T:82 (68)	T:51	Spartalizumab (PDR001) 400 mg Q4W	28	T:25.2	T:1.9	T:18.4	Liver; lung	T:78	NR
			C:40 (33)	C:50			C:15.5	C:6.6	C:32.5		C:38	
Single-armed experiment												
P Economopoulou	2022	PD-1	46 (36)	56.3	Nivolumab/pembrolizumab	60	19.1	5.6	26.2	Liver; lung; bone	NR	II-IV
WF Fang	2018	PD-1	93 (75)	45	Camrelizumab 3 mg/kg Q2W	12	NR	5.6	34	Liver; lung	NR	NR
C Hus	2017	PD-1	27 (21)	52	Pembrolizumab 10 mg/kg Q2W	28	16.5	6.5	25.9	Liver; lung; bone; Lymph node	41	II-III
BBY Ma	2018	PD-1	45 (35)	57	Nivolumab 3 mg/kg Q2W	24	17.1	2.8	20.5	Liver; lung; bone; Lymph node	18	NR
YX Ma	2021	PD-1	124 (95)	46	Camrelizumab 10 mg/kg Q2W/Nivolumab 3 mg/kg Q3W	34	17.1	3.8	29.8	Liver; lung; bone; Lymph node	NR	NR
H Sato	2020	PD-1	12 (10)	58	Nivolumab 3 mg/kg Q2W	20	NR	3.6	16.7	Liver; lung; bone; Lymph node	1	II-IVC
FH Wang	2021	PD-1	190 (158)	46.4	Toripalimab 3 mg/kg Q2W	40	17.4	1.9	20.5	Liver; lung; bone; Lymph node	48	III-IVb

Abbreviation: T, treatment group; C, control group; GP, gemcitabine-cisplatin; MO, months; OS, overall survival; PFS, progression free survival; ORR, objective response rate; NR, not reported; NRE, not reached; Q3W, every 3 weeks.

### 3.3 Meta analysis for RCT

#### 3.3.1 ORR

**FIGURE 2:**
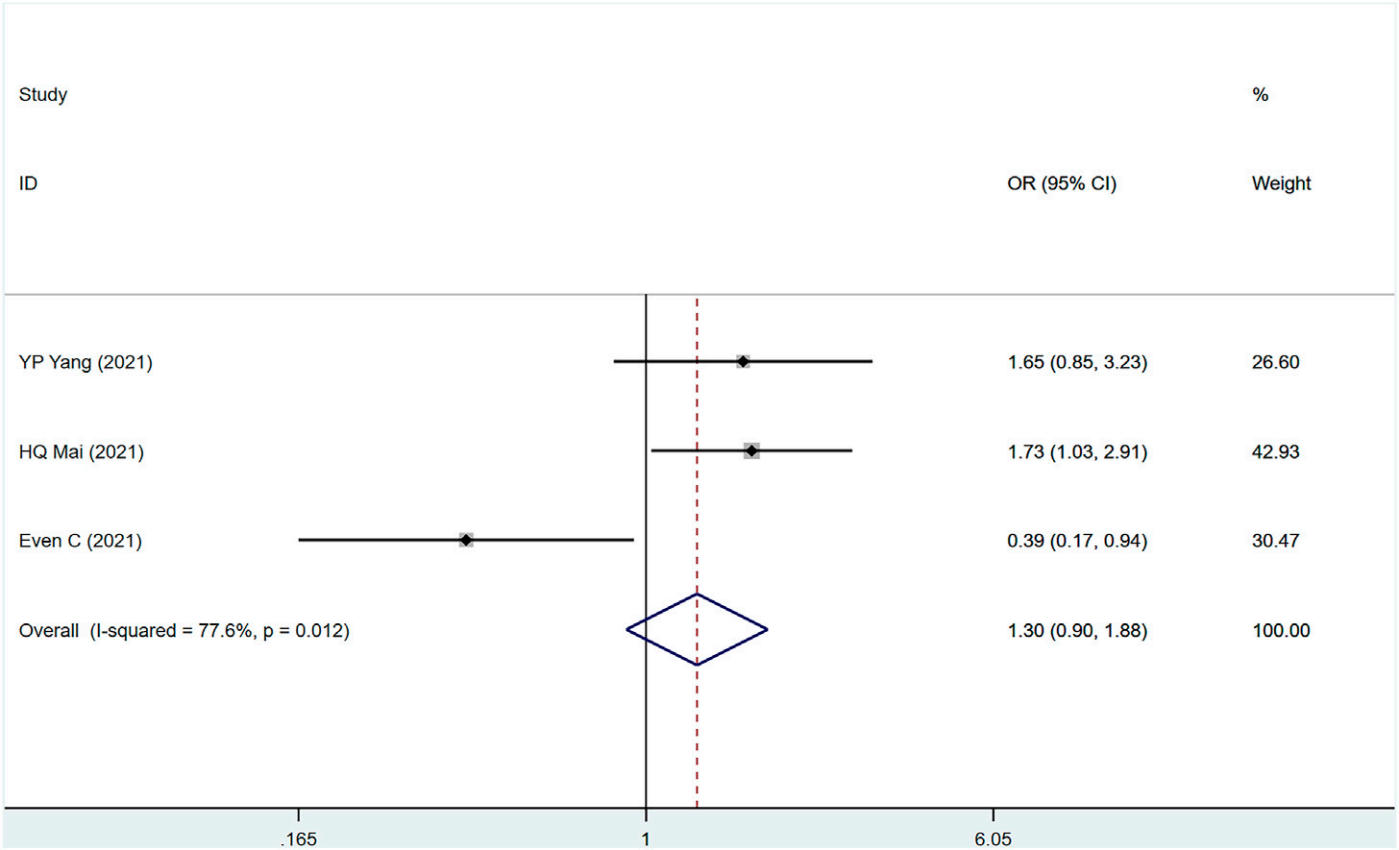
Estimated ORR proportion (95% CI) of patients with metastatic/recurrent nasopharyngeal carcinoma after PD-1 treatment forest plot—randomized controlled trial.

A total of three studies involved a total of 672 patients with metastatic/recurrent NPC. There were 360 patients in the PD-1 inhibitor group and 312 patients in the control group (I2 = 77.6%, *p* = .012), indicating higher heterogeneity. Figure 2 [OR = 1.11, 95% CI (.49, 2.52); *p* = .812] suggested that PD-1 inhibitors did not improve ORR in patients with metastatic/recurrent NPC. Sensitivity analysis was performed on the deleted studies one by one, and it was found that the potential heterogeneity may originate from Even C (23) (Supplementary Figure S2A). *p*-values for assessing publication bias (Egger test: .294, Begg test: .117) were all >.05, indicating that there is a small possibility that there is no publication bias (Supplementary Table S4).

#### 3.3.2 OS

**FIGURE 3:**
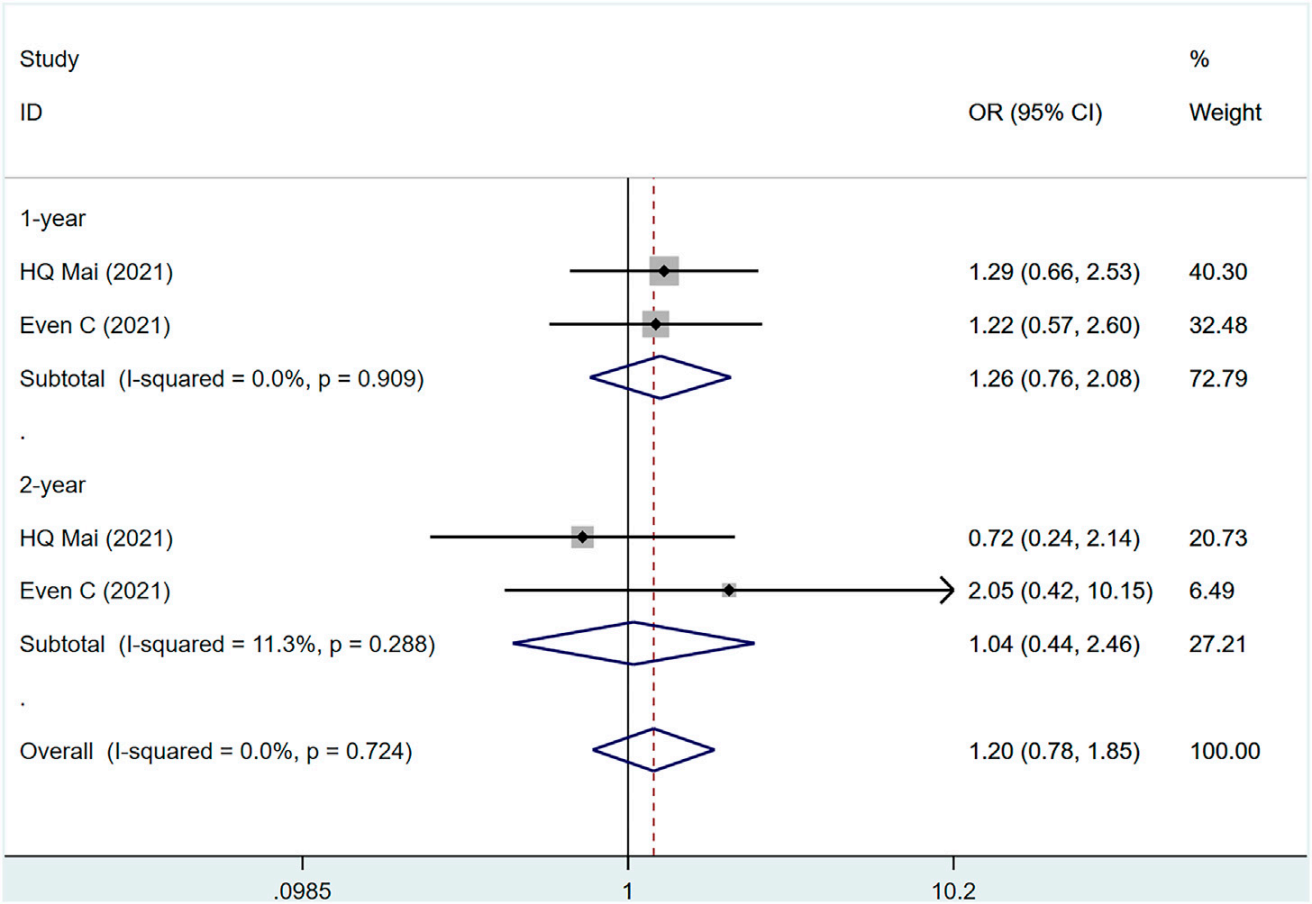
Estimated OS proportion (95% CI) of patients with metastatic/recurrent nasopharyngeal carcinoma after PD-1 treatment forest plot—randomized controlled trial.

Two studies (Even et al., 2021; Mai et al., 2021) involved a total of 441 patients, of which 228 were PD-1 and 183 were control group (I2 = 0%, *p* = .724), suggesting that the heterogeneity is acceptable. Figure 3 shows that PD-1 inhibitors had no effect on 1- and 2-year OS in patients with metastatic/recurrent NPC [1-year OR = 1.26, 95% CI (.76, 2.08); *p* = .367]; [2-year OR = 1.03, 95% CI (.39, 2.71); *p* = .928]. *p*-values for assessing publication bias (Egger test: .718, Begg test: 1.000) were all >.05, indicating that there is a small possibility that there is no publication bias (Supplementary Table S4).

#### 3.3.3 PFS

**FIGURE 4:**
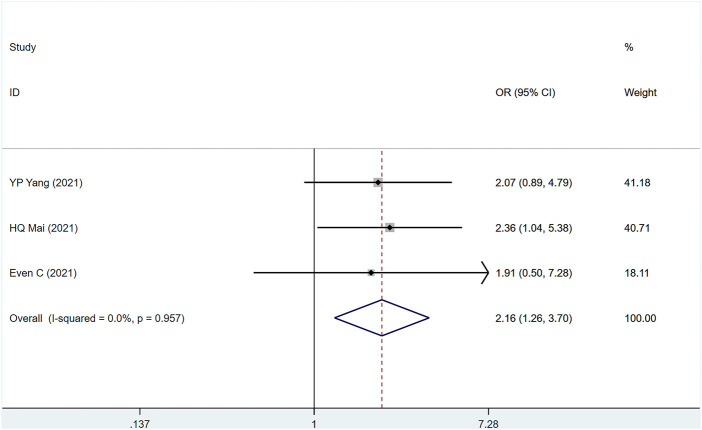
Estimated PFS proportion (95% CI) of patients with metastatic/recurrent nasopharyngeal carcinoma after PD-1 treatment forest plot—randomized controlled trial.

The three studies (Even et al., 2021; Mai et al., 2021; Yang et al., 2021) involved a total of 604 people, of which 313 were PD-1 and 291 were control group. (I2 = 0%, *p* = .957), suggesting that the heterogeneity is small. Figure 4 [OR = 2.16, 95% CI (1.26, 3.70); *p* = .005]; suggests that PD-1 inhibitors can improve the 1-year PFS of metastatic/recurrent NPC patients. *p*-values for assessing publication bias (Egger test: .528, Begg test: 1.040) were all >.05, indicating that there is a small possibility that there is no publication bias (Supplementary Table S4).

### 3.4 Meta analysis for single-arm study

#### 3.4.1 ORR

**FIGURE 5:**
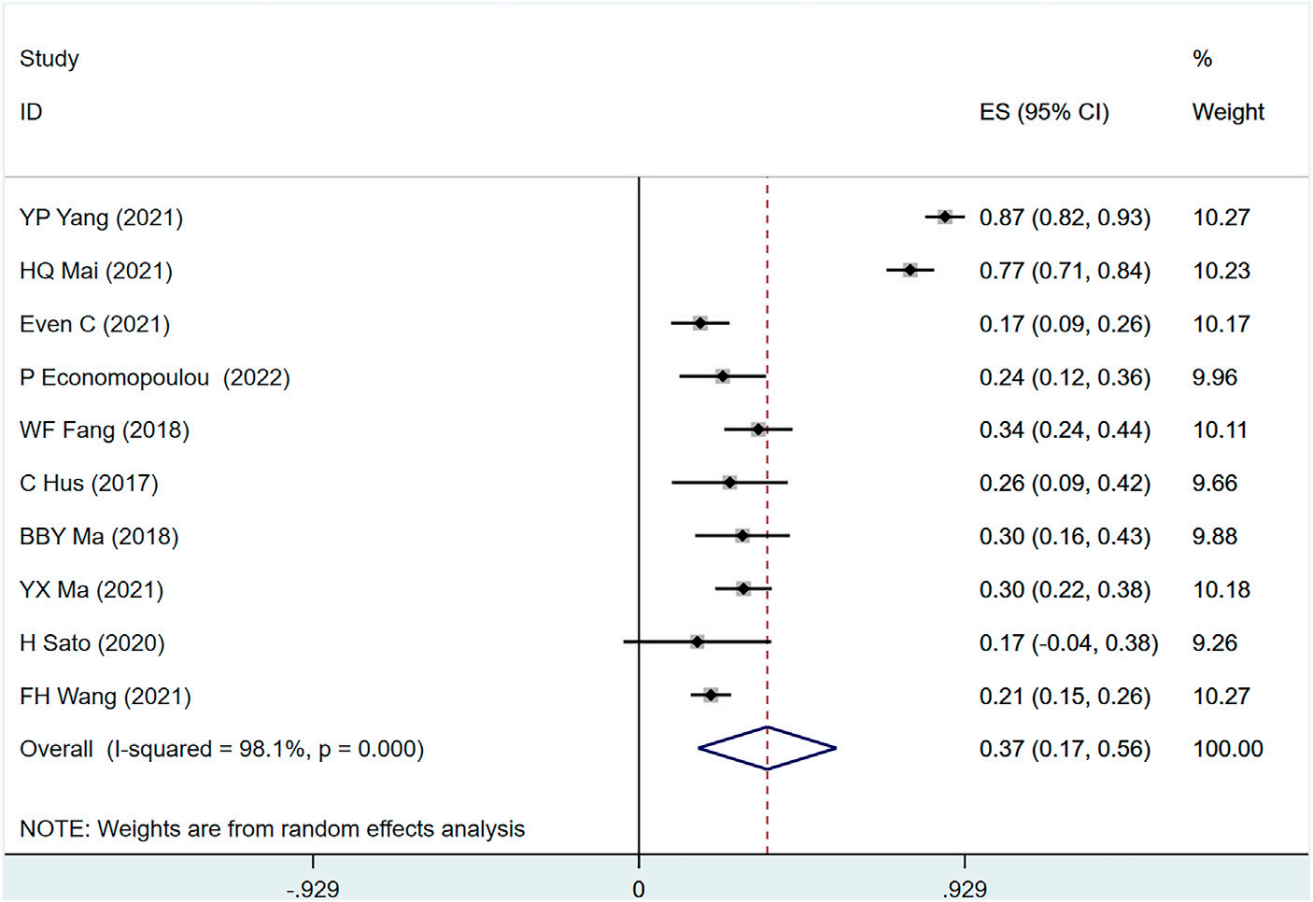
Estimated ORR proportion (95% CI) of patients with metastatic/recurrent nasopharyngeal carcinoma after PD-1 treatment forest plot—single arm studies.

ORR was mentioned in 10 studies (Hsu et al., 2017; Fang et al., 2018; Ma et al., 2018; Sato et al., 2020; Even et al., 2021; Ma et al., 2021; Mai et al., 2021; Wang et al., 2021; Yang et al., 2021; Economopoulou et al., 2022) involving 894 people, (I2 = 98.1, *p* = 0) suggesting a large heterogeneity among the included studies. Figure 5 shows ORR in metastatic/recurrent NPC patients treated with PD-1 inhibitors [ES = 37%, 95% CI (17%–56%); *p* = .00]. Sensitivity analysis indicated that the analysis results were relatively stable (Supplementary Figure S2B), with *p* > .05 for Egger’s and Begg’s publication bias assessment (Egger test: .244, Begg test: 0.180), suggesting that there was a small possibility of publication bias (Supplementary Table S4).

#### 3.4.2 OS

**FIGURE 6:**
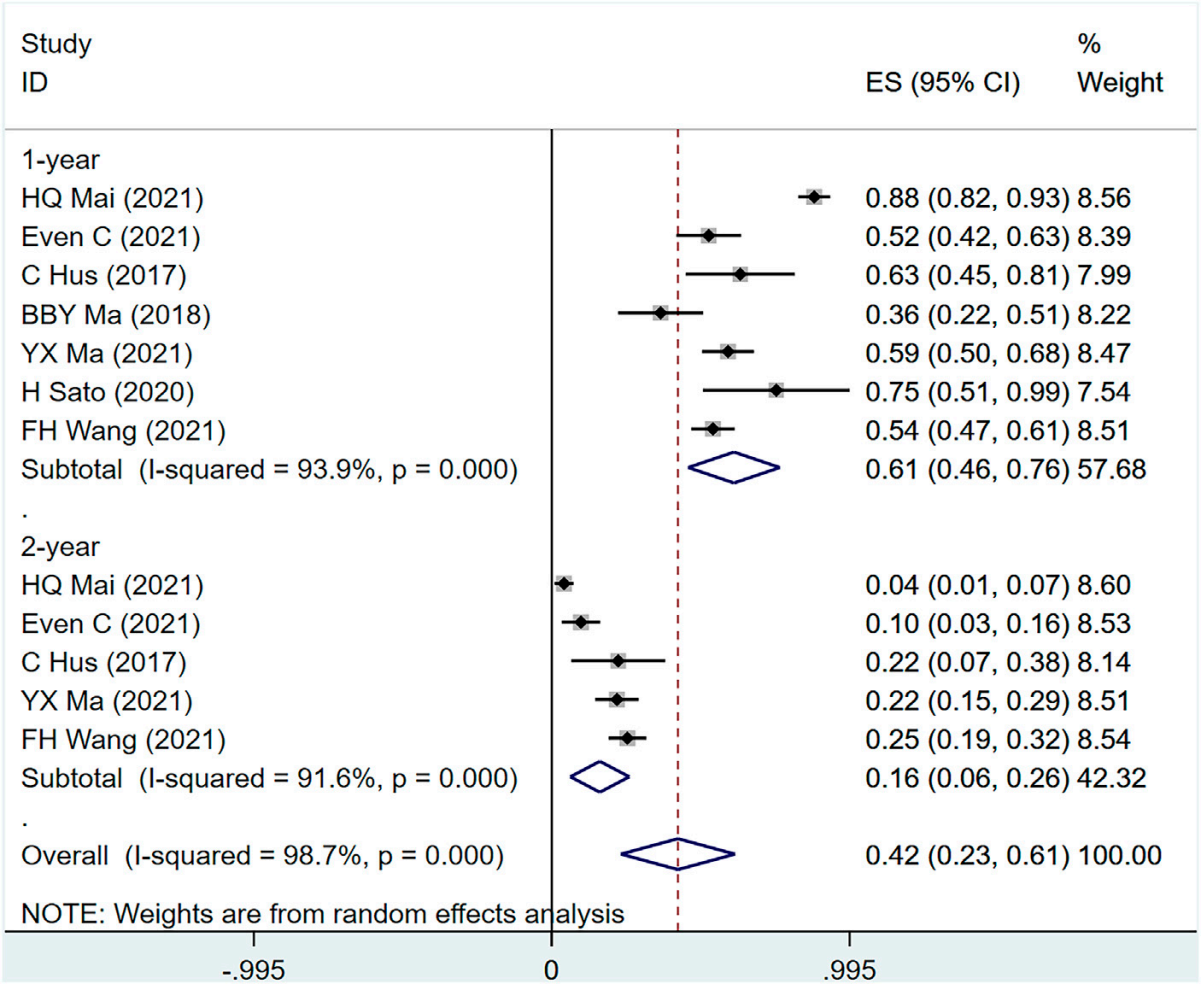
Estimated OS proportion (95% CI) of patients with metastatic/recurrent nasopharyngeal carcinoma after PD-1 treatment forest plot—single arm studies.

OS was mentioned in seven studies including 617 people (I2 = 98.7, *p* = 0), indicating that the heterogeneity among included studies was large. Figure 6 shows the OS of patients with metastatic/recurrent NPC using PD-1 inhibitors [1-year ES = 61%, 95% CI (46%–76%); *p* = .00]; [2-year ES = 16%, 95% CI (6%–26%); *p* = .001]. Sensitivity analysis suggests that the analysis results are relatively stable (Supplementary Figure S2C), with *p* > .05 for the evaluation of egg’s and Begg’s in publication bias (Egger test: .196, Begg test: .583), suggesting the possibility of publication bias Less sexual (Supplementary Table S4).

#### 3.4.3 PFS

**FIGURE 7:**
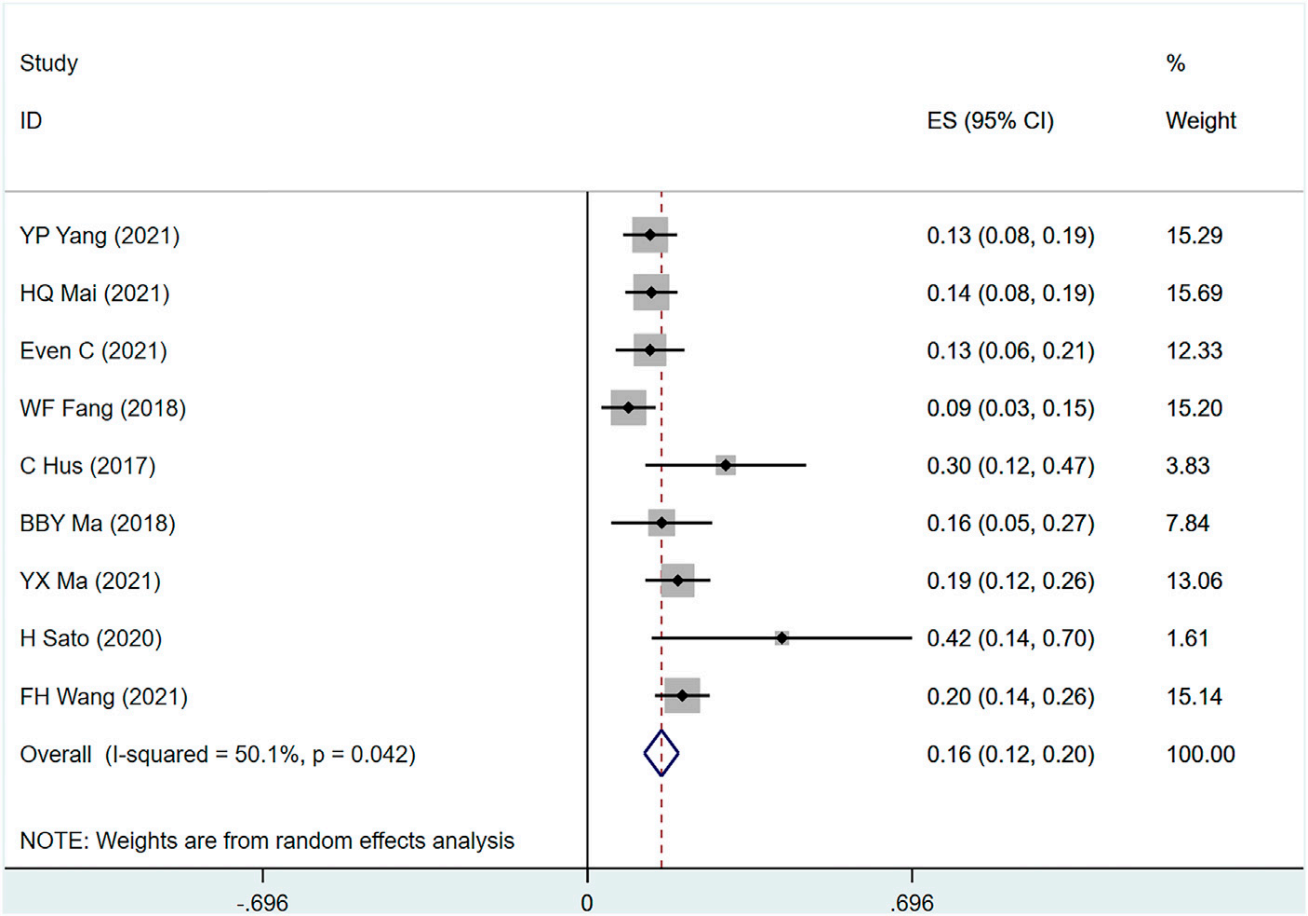
Estimated PFS proportion (95% CI) of patients with metastatic/recurrent nasopharyngeal carcinoma after PD-1 treatment forest plot—single arm studies.

PFS was mentioned in nine studies (Hsu et al., 2017; Fang et al., 2018; Ma et al., 2018; Sato et al., 2020; Even et al., 2021; Ma et al., 2021; Mai et al., 2021; Wang et al., 2021; Yang et al., 2021) involving 842 people, (I2 = 50.1.7, *p* = .042) suggesting the heterogeneity among included studies. Figure 7 shows the use of PD-1 inhibitors for PFS in patients with metastatic/recurrent NPC [1-year ES = 16%, 95% CI (12%–20%); *p* = .00]. Sensitivity analysis indicated that the analysis results were relatively stable (Supplementary Figure S2D), with *p* > .05 for Egger’s and Begg’s (Egger test: .074, Begg test: .095), suggesting that there is a small possibility of publication bias (Supplementary Table S4).

### 3.5 Meta analysis for adverse event

Among the 10 included studies (Hsu et al., 2017; Fang et al., 2018; Ma et al., 2018; Sato et al., 2020; Even et al., 2021; Ma et al., 2021; Mai et al., 2021; Wang et al., 2021; Yang et al., 2021; Economopoulou et al., 2022), the main adverse events were rash, Leukopenia, Anemia, Neutropenia, Vomiting, Thrombocytopenia, Decreased appetite, and Constipation. In any grade ES (rash = 18%, Leukopenia = 66%, Anemia = 36%, Neutropenia = 63%, Vomiting = 62%, Thrombocytopenia = 48%, Reduced appetite = 40%, Constipation = 28%), in grade ≥3 ES (rash = 2%, Leukopenia = 64%, Anemia = 18%, Neutropenia = 31%, Vomiting = 4%, Thrombocytopenia = 24%, and Reduced appetite = 1%) (Supplementary Table S5).

### 3.6 Survival curve analysis

**FIGURE 8:**
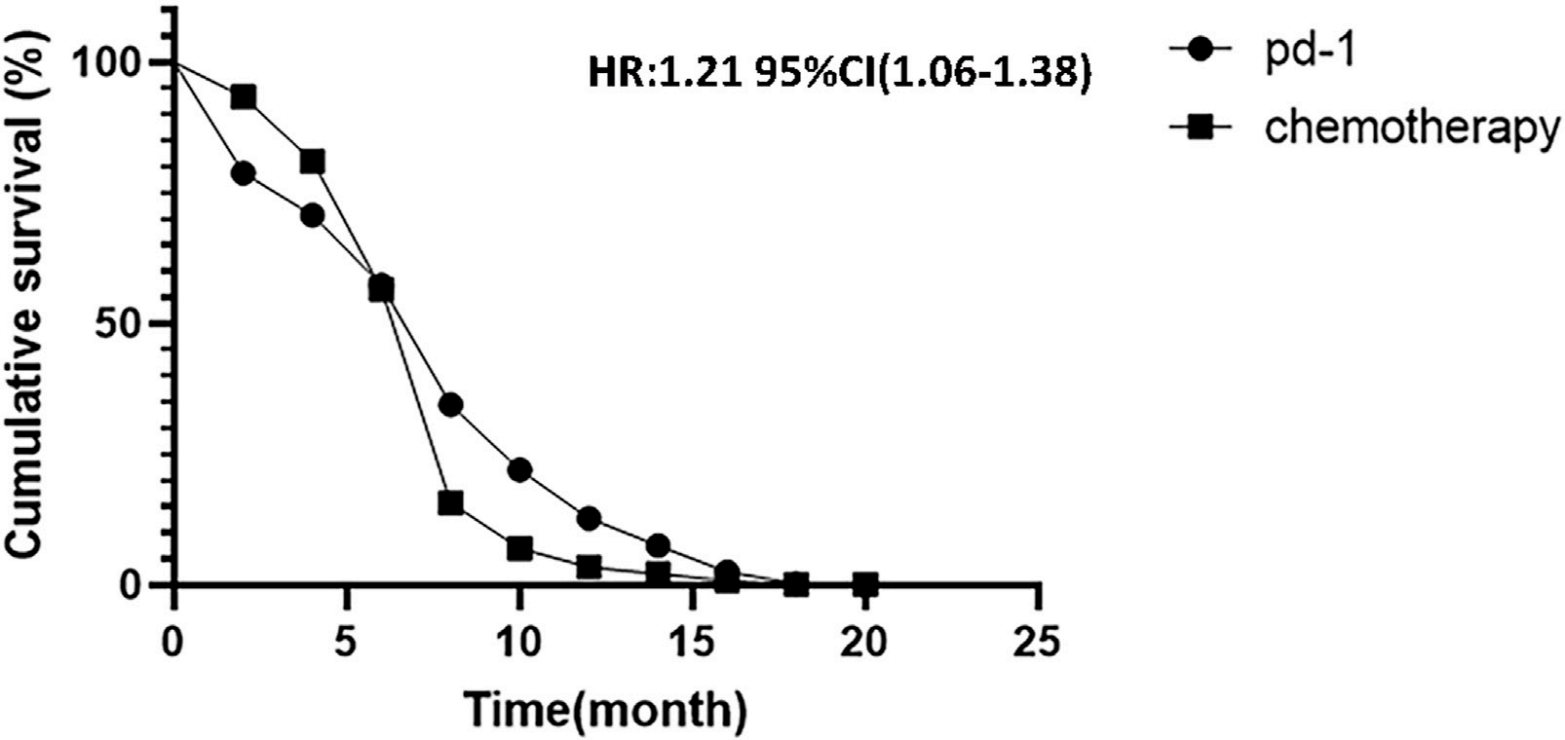
Kaplan-Meier analysis of cumulative progression-free survival between PD-1 inhibitors and chemotherapy.

Combined with the PFS of three randomized controlled trials, the survival curve is shown in Figure 8 [HR = 1.21, 95% CI (1.06, 1.38), shown in Figure 8. *p* = .002]. This indicates that PD-1 inhibitor showed a significant extension of PFS in patients with PD-1 inhibitor compared to traditional chemotherapy.

## 4 Discussion

As far as we know, this is not the first meta-analysis on similar topics. Wang et al. (2020) published a similar meta-paper in 2020, but compared with Wang, this study has the following advantages: first, the latest RCTs and single-arm studies were included, and the latest evidence was obtained; second, most of the articles included were conference articles and case reports, and no obvious conclusions were drawn in Wang’s paper. Therefore, compared with this paper, our paper has obvious advantages and innovation.

Once the tumor metastasizes, it means that the disease changes from a local disease to a systemic disease (Klein, 2009). The clinical treatment is mainly palliative chemotherapy. However, due to the previous treatment usage of multiple chemotherapeutic medications, drug sensitivity is reduced, and treatment effect is frequently poor (Prada et al., 2013). Currently, PD-1 is believed to be expressed on activated T cells, B cells, NK cells, and macrophages. Therefore, the use of PD-1 antibodies for the treatment of metastatic/recurrent NPC can enhance the body’s immune system. The way of anti-tumor effect, synergistic chemotherapy enhances the therapeutic effect (Kim and Chen, 2016).

For meta-analysis of RCTs, we found that ORR [OR = 1.11, 95% CI (.49, 2.52); *p* = .812], 1-year OS [OR = 1.26, 95% CI (.76, 2.08); *p* = .367], and 2-year OS [OR = 1.03, 95% CI (.39, 2.71); *p* = .928] in patients with metastatic/recurrent NPC were consistent with PD-1 and conventional chemotherapy. This is the first RCT-based conclusion on PD-1 inhibitor therapy for relapsed/metastatic NPC, but we should treat the conclusion with caution because the number of included studies was small, the included study drug was not the same, and the treatment of Even C (23) only used PD-1 inhibitors, while the other two studies used Gemcitabine-cisplatin. However, PD-1 inhibitors outperformed conventional chemotherapy in terms of 1-year PFS [OR = 2.16, 95% CI (1.26, 3.70); *p* = .005]. Possible explanations include: I) some patients receiving PD-1 inhibitor treatment for a long time after the therapeutic impact was determined to have an advanced disease; and II) some patients receiving follow-up treatment for a longer period after receiving PD-1 inhibitor therapy (Xie et al., 2019; Jiang et al., 2021). With the results of multiple phase III trials (CAPTAIN first, JUPITER 02, and RATIONALE 309), RATIONALE-309 (Zhang et al., 2022) is consistent with the conclusion. 263 eligible metastatic/recurrent NPC patients were randomly assigned 1:1 to receive tislelizumab 200 mg IV or placebo. Results showed that, compared with placebo + chemotherapy, Tislelizumab plus chemotherapy showed a consistent, clinically meaningful improvement in PFS. We believe that PD-1 inhibitors therapy can achieve a breakthrough in the treatment of recurrent or metastatic NPC (Hua et al., 2022; Huang et al., 2022).

For the meta-analysis of single-arm studies, after PD-1 inhibitors therapy, the ORR of patients with recurrent/metastatic NPC reached [ES = 37%, 95% CI (17%–56%)], 1-year OS [ES = 61%, 95% CI (46%–76%)], 2-year [ES = 16%, 95% CI (6%–26%)], and 1-year PFS [ES = 16%, 95% CI (12%–20%)]. This is consistent with the conclusion drawn by Lin and colleagues (Lin et al., 2022). The 12 patients with conventional chemotherapy + PD-1 inhibitors had an overall best ORR of 66.6% and a disease control rate (DCR) as high as 66.6%, the OS in June and December was 87.5% and 63.5%, respectively, and the median OS was not reached. This result suggests that conventional chemotherapy + PD-1 inhibitors treatment can effectively improve the remission rate even if the tumor progresses after PD-1 treatment, and strengthens the evidence that palliative chemotherapy participates in combined immunotherapy after PD-1 resistance. The advantages of combined chemotherapy in cancer were comparable to immune monotherapy. Possible reasons: 1. The combination of chemotherapy and PD-1 inhibitor therapy has a synergistic effect. Chemotherapy can transform “cold tumors” into “hot tumors” by changing the tumor microenvironment, thereby increasing the efficacy of immunotherapy (Herrera et al., 2017). 2. NPC itself is immunogenic, and EBV-infected nasopharyngeal carcinoma cells express target proteins of CD4^+^ T cells and CD8^+^ T cells. 3. Combination of the chemotherapy and PD-1 antibody can significantly increase the degree of tumor CTL infiltration and reduce the level of regulatory T cells, thereby significantly prolonging the survival time (Jarzab et al., 2005). The PACIFIC study (Antonia et al., 2017) enrolled 713 patients. In a 2:1 randomization, it was found that use of Durvalumab consolidation therapy after concurrent chemotherapy significantly prolonged the progression-free survival of these patients (17.2 months vs. 5.6 months). This revolutionized the field of radiotherapy combined with immunization. Therefore, we believe that the combined treatment mode of radiotherapy and anti-PD-1 still has clinical practical significance for recurrent and metastatic NPC patients with immune resistance (Theodoraki et al., 2022; Tian et al., 2022). The frequency of associated adverse events, such as nausea, vomiting, and anemia, is not increased in patients with recurrent/metastatic NPC treated with PD-1 inhibitors. The majority of the adverse effects were tolerable in grades 1–2 and may be reduced with appropriate therapy. Thus, adverse effects do not preclude the use of PD-1 inhibitors in metastatic/recurrent NPC (Yang et al., 2022).

There are still some limitations to this study. First: the number of included studies is small, and there are few randomized controlled trials, so there may be an influence on the conclusion. Second: the drug dosage and frequency of drugs used in the included studies were not consistently included in the studies are not consistent. Some studies used combination drugs, but some studies used drugs alone, which may lead to a large potential heterogeneity between studies. Third, despite doing an extensive systematic search of topics and a manual search of relevant articles to retrieve as many eligible papers as possible, it is likely that relevant studies were missed or excluded.

## 5 Conclusion

According to the available evidence, the efficacy of PD-1 inhibitor monotherapy in patients with metastatic/recurrent nasopharyngeal carcinoma was not significantly different from that of conventional chemotherapy; however, due to the limitations of the included studies, further phase III RCTs are required to corroborate our conclusion.

## Data Availability

The original contributions presented in the study are included in the article/Supplementary Material, further inquiries can be directed to the corresponding author.

## References

[B1] Antonia S. J. Villegas A. Daniel D. Vicente D. Murakami S. Hui R. (2017). Durvalumab after chemoradiotherapy in stage III non-small-cell lung cancer. N. Engl. J. Med. 377 (20), 1919–1929. 10.1056/NEJMoa1709937 28885881

[B2] Beyene E. T. Ketema S. G. Alebachew A. N. Saleh M. Y. Gebremariam T. A. (2021). Descriptive epidemiology of nasopharyngeal carcinoma at tikur anbessa hospital, Ethiopia. BMC Cancer 21 (1), 540. 10.1186/s12885-021-08311-8 33980204 PMC8114688

[B3] Bossi P. Chan A. T. Licitra L. Trama A. Orlandi E. Hui E. P. (2021). Nasopharyngeal carcinoma: ESMO-EURACAN clinical practice guidelines for diagnosis, treatment and follow-up(†). Ann. Oncol. 32 (4), 452–465. 10.1016/j.annonc.2020.12.007 33358989

[B4] Bray F. Ferlay J. Soerjomataram I. Siegel R. L. Torre L. A. Jemal A. (2018). Global cancer statistics 2018: GLOBOCAN estimates of incidence and mortality worldwide for 36 cancers in 185 countries. CA Cancer J. Clin. 68 (6), 394–424. 10.3322/caac.21492 30207593

[B5] Chang E. T. Ye W. Zeng Y. X. Adami H. O. (2021). The evolving epidemiology of nasopharyngeal carcinoma. Cancer Epidemiol. Biomarkers Prev. 30 (6), 1035–1047. 10.1158/1055-9965.EPI-20-1702 33849968

[B6] Chen Y. P. Chan A. T. C. Le Q. T. Blanchard P. Sun Y. Ma J. (2019). Nasopharyngeal carcinoma. Lancet 394 (10192), 64–80. 10.1016/S0140-6736(19)30956-0 31178151

[B7] Diesendruck Y. Benhar I. (2017). Novel immune check point inhibiting antibodies in cancer therapy-Opportunities and challenges. Drug Resist Updat 30, 39–47. 10.1016/j.drup.2017.02.001 28363334

[B8] Dwijayanti F. Prabawa A. Besral Herawati C. (2020). The five-year survival rate of patients with nasopharyngeal carcinoma based on tumor response after receiving neoadjuvant chemotherapy, followed by chemoradiation, in Indonesia: A retrospective study. Oncology 98 (3), 154–160. 10.1159/000504449 31995803

[B9] Economopoulou P. Pantazopoulos A. Spathis A. Kotsantis I. Kyriazoglou A. Kavourakis G. (2022). Immunotherapy in nonendemic nasopharyngeal carcinoma: Real-world data from two nonendemic regions. Cells 11 (1), 32. 10.3390/cells11010032 PMC8750043 35011594

[B10] Even C. Wang H. M. Li S. H. Ngan R. K. Dechaphunkul A. Zhang L. (2021). Phase II, randomized study of spartalizumab (PDR001), an anti-PD-1 antibody, versus chemotherapy in patients with recurrent/metastatic nasopharyngeal cancer. Clin. Cancer Res. 27 (23), 6413–6423. 10.1158/1078-0432.CCR-21-0822 34433653

[B11] Fang W. Yang Y. Ma Y. Hong S. Lin L. He X. (2018). Camrelizumab (SHR-1210) alone or in combination with gemcitabine plus cisplatin for nasopharyngeal carcinoma: Results from two single-arm, phase 1 trials. Lancet Oncol. 19 (10), 1338–1350. 10.1016/S1470-2045(18)30495-9 30213452

[B12] Gohil S. H. Iorgulescu J. B. Braun D. A. Keskin D. B. Livak K. J. (2021). Applying high-dimensional single-cell technologies to the analysis of cancer immunotherapy. Nat. Rev. Clin. Oncol. 18 (4), 244–256. 10.1038/s41571-020-00449-x 33277626 PMC8415132

[B13] Herrera F. G. Bourhis J. Coukos G. (2017). Radiotherapy combination opportunities leveraging immunity for the next oncology practice. CA Cancer J. Clin. 67 (1), 65–85. 10.3322/caac.21358 27570942

[B14] Hiam-Galvez K. J. Allen B. M. Spitzer M. H. (2021). Systemic immunity in cancer. Nat. Rev. Cancer 21 (6), 345–359. 10.1038/s41568-021-00347-z 33837297 PMC8034277

[B15] Hsu C. Lee S. H. Ejadi S. Even C. Cohen R. B. Le Tourneau C. (2017). Safety and antitumor activity of Pembrolizumab in patients with programmed death-ligand 1-positive nasopharyngeal carcinoma: Results of the KEYNOTE-028 study. J. Clin. Oncol. 35 (36), 4050. 10.1200/JCO.2017.73.3675 28837405

[B16] Hua Y. H. Dong R. Z. Jin T. Jin Q. F. Chen X. Z. (2022). Anti-PD-1 monoclonal antibody combined with anti-VEGF agent is safe and effective in patients with recurrent/metastatic head and neck squamous cancer as second-line or beyond treatment. Front. Oncol. 12. 10.3389/fonc.2022.781348 PMC8908371 35280787

[B17] Huang J. L. Chen S. Y. Lin C. S. (2022). Targeting cancer stem cells through epigenetic modulation of interferon response. J. Personalized Med. 12 (4), 556. 10.3390/jpm12040556 PMC9027081 35455671

[B18] Jarzab B. Handkiewicz-Junak D. Wloch J. (2005). Juvenile differentiated thyroid carcinoma and the role of radioiodine in its treatment: A qualitative review. Endocr. Relat. Cancer 12 (4), 773–803. 10.1677/erc.1.00880 16322322

[B19] Jiang W. Pan S. Chen X. Wang Z. W. Zhu X. (2021). The role of lncRNAs and circRNAs in the PD-1/PD-L1 pathway in cancer immunotherapy. Mol. Cancer 20 (1), 116. 10.1186/s12943-021-01406-7 34496886 PMC8424797

[B20] Kang Y. He W. Ren C. Qiao J. Guo Q. Hu J. (2020). Advances in targeted therapy mainly based on signal pathways for nasopharyngeal carcinoma. Signal Transduct. Target Ther. 5 (1), 245. 10.1038/s41392-020-00340-2 33093441 PMC7582884

[B21] Kim J. M. Chen D. S. (2016). Immune escape to PD-L1/PD-1 blockade: Seven steps to success (or failure). Ann. Oncol. 27 (8), 1492–1504. 10.1093/annonc/mdw217 27207108

[B22] Klein C. A. (2009). Parallel progression of primary tumours and metastases. Nat. Rev. Cancer 9 (4), 302–312. 10.1038/nrc2627 19308069

[B23] Kumar P. Saini S. Prabhakar B. S. (2020). Cancer immunotherapy with check point inhibitor can cause autoimmune adverse events due to loss of Treg homeostasis. Semin. Cancer Biol. 64, 29–35. 10.1016/j.semcancer.2019.01.006 30716481

[B24] Lee A. W. Ma B. B. Ng W. T. Chan A. T. (2015). Management of nasopharyngeal carcinoma: Current practice and future perspective. J. Clin. Oncol. 33 (29), 3356–3364. 10.1200/JCO.2015.60.9347 26351355

[B25] Lee H. M. Okuda K. S. González F. E. Patel V. (2019). Current perspectives on nasopharyngeal carcinoma. Adv. Exp. Med. Biol. 1164, 11–34. 10.1007/978-3-030-22254-3_2 31576537

[B26] Li X. Wenes M. Romero P. Huang S. C. Fendt S. M. Ho P. C. (2019). Navigating metabolic pathways to enhance antitumour immunity and immunotherapy. Nat. Rev. Clin. Oncol. 16 (7), 425–441. 10.1038/s41571-019-0203-7 30914826

[B27] Lin J. Guo Q. Guo Z. Lu T. Chen G. Lin S. (2022). Stereotactic body radiotherapy extends the clinical benefit of PD-1 inhibitors in refractory recurrent/metastatic nasopharyngeal carcinoma. Radiat. Oncol. 17 (1), 117. 10.1186/s13014-022-02073-8 35790987 PMC9254565

[B28] Lo C. K. Mertz D. Loeb M. (2014). Newcastle-ottawa Scale: Comparing reviewers' to authors' assessments. BMC Med. Res. Methodol. 14, 45. 10.1186/1471-2288-14-45 24690082 PMC4021422

[B29] Ma B. B. Y. Lim W. T. Goh B. C. Hui E. P. Lo K. W. Pettinger A. (2018). Antitumor activity of nivolumab in recurrent and metastatic nasopharyngeal carcinoma: An international, multicenter study of the mayo clinic phase 2 consortium (NCI-9742). J. Clin. Oncol. 36 (14), 1412–1418. 10.1200/JCO.2017.77.0388 29584545 PMC5941615

[B30] Ma Y. Chen X. Wang A. Zhao H. Lin Q. Bao H. (2021). Copy number loss in granzyme genes confers resistance to immune checkpoint inhibitor in nasopharyngeal carcinoma. J. Immunother. Cancer 9 (3), e002014. 10.1136/jitc-2020-002014 33737344 PMC7978327

[B31] Mai H. Q. Chen Q. Y. Chen D. P. Hu C. S. Yang K. Y. Wen J. Y. (2021). Toripalimab or placebo plus chemotherapy as first-line treatment in advanced nasopharyngeal carcinoma: A multicenter randomized phase 3 trial. Nat. Med. 27 (9), 1536–1543. 10.1038/s41591-021-01444-0 34341578

[B32] Prada C. E. Jousma E. Rizvi T. A. Wu J. Dunn R. S. Mayes D. A. (2013). Neurofibroma-associated macrophages play roles in tumor growth and response to pharmacological inhibition. Acta Neuropathol. 125 (1), 159–168. 10.1007/s00401-012-1056-7 23099891 PMC3547628

[B33] Riley R. S. June C. H. Langer R. Mitchell M. J. (2019). Delivery technologies for cancer immunotherapy. Nat. Rev. Drug Discov. 18 (3), 175–196. 10.1038/s41573-018-0006-z 30622344 PMC6410566

[B34] Sato H. Fushimi C. Okada T. Matsuki T. Kondo T. Omura G. O. (2020). Investigation of the efficacy and safety of nivolumab in recurrent and metastatic nasopharyngeal carcinoma. Vivo 34 (5), 2967–2972. 10.21873/invivo.12127 PMC7652473 32871839

[B35] Sterne J. A. C. Savović J. Page M. J. Elbers R. G. Blencowe N. S. Boutron I. (2019). RoB 2: A revised tool for assessing risk of bias in randomised trials. Bmj 366, l4898. 10.1136/bmj.l4898 31462531

[B36] Sun X. S. Li X. Y. Chen Q. Y. Tang L. Q. Mai H. Q. (2019). Future of radiotherapy in nasopharyngeal carcinoma. Br. J. Radiol. 92 (1102), 20190209. 10.1259/bjr.20190209 31265322 PMC6774595

[B37] Theodoraki M. N. Laban S. Hoffmann T. K. (2022). Immunotherapy of head and neck cancer Highlights of the ASCO and ESMO annual meetings 2021. Hno 70 (4), 271–277. 10.1007/s00106-021-01142-w 35037989

[B38] Tian K. Han J. Q. Wang Z. Chen J. (2022). Immune checkpoint inhibition in first-line treatment for recurrent or metastatic nasopharyngeal carcinoma: A CAPTAIN-1st and JUPITER-02 trial-based cost-effectiveness analysis. Oral Oncol. 128, 105842. 10.1016/j.oraloncology.2022.105842 35428025

[B39] Tsang C. M. Lui V. W. Y. Bruce J. P. Pugh T. J. Lo K. W. (2020). Translational genomics of nasopharyngeal cancer. Semin. Cancer Biol. 61, 84–100. 10.1016/j.semcancer.2019.09.006 31521748

[B40] Wang B. C. Cao R. B. Fu C. Chen W. B. Li P. D. Lin G. H. (2020). The efficacy and safety of PD-1/PD-L1 inhibitors in patients with recurrent or metastatic nasopharyngeal carcinoma: A systematic review and meta-analysis. Oral Oncol. 104, 104640. 10.1016/j.oraloncology.2020.104640 32182550

[B41] Wang F. H. Wei X. L. Feng J. Li Q. Xu N. Hu X. C. (2021). Efficacy, safety, and correlative biomarkers of Toripalimab in previously treated recurrent or metastatic nasopharyngeal carcinoma: A phase II clinical trial (POLARIS-02). J. Clin. Oncol. 39 (7), 704–712. 10.1200/JCO.20.02712 33492986 PMC8078488

[B42] Xie F. Xu M. Lu J. Mao L. Wang S. (2019). The role of exosomal PD-L1 in tumor progression and immunotherapy. Mol. Cancer 18 (1), 146. 10.1186/s12943-019-1074-3 31647023 PMC6813045

[B43] Yang J. Chen J. Liang H. Yu Y. (2022). Nasopharyngeal cancer cell-derived exosomal PD-L1 inhibits CD8+ T cell activity and promotes immune escape. Cancer Sci. 113, 3044–3054. 10.1111/cas.15433 35598173 PMC9459270

[B44] Yang Y. P. Zhou T. Chen X. Z. Li J. A. Pan J. J. He X. H. (2021). Efficacy, safety, and biomarker analysis of Camrelizumab in previously treated recurrent or metastatic nasopharyngeal carcinoma (CAPTAIN study). J. Immunother. Cancer 9 (12), e003790. 10.1136/jitc-2021-003790 34933967 PMC8693086

[B45] Yarza R. Bover M. Agulló-Ortuño M. T. Iglesias-Docampo L. C. (2021). Current approach and novel perspectives in nasopharyngeal carcinoma: The role of targeting proteasome dysregulation as a molecular landmark in nasopharyngeal cancer. J. Exp. Clin. Cancer Res. 40 (1), 202. 10.1186/s13046-021-02010-9 34154654 PMC8215824

[B46] Zhang L. Yang Y. Pan J. Chen X. Sun Y. Wang H. (2022). RATIONALE-309: Updated progression-free survival (PFS), PFS after next line of treatment, and overall survival from a phase 3 double-blind trial of tislelizumab versus placebo, plus chemotherapy, as first-line treatment for recurrent/metastatic nasopharyngeal cancer. J. Clin. Oncol. 40 (36), 384950. 10.1200/jco.2022.40.36_suppl.384950

[B47] Zhang Y. Chen L. Hu G. Q. Zhang N. Zhu X. D. Yang K. Y. (2019). Gemcitabine and cisplatin induction chemotherapy in nasopharyngeal carcinoma. N. Engl. J. Med. 381 (12), 1124–1135. 10.1056/NEJMoa1905287 31150573

