# Supplementary material for: Are PD-1 inhibitors effective for recurrent/metastatic nasopharyngeal carcinoma? Meta-analysis and systematic review
Source: Front Pharmacol. 2023 Jan 9;13:1095734. doi: 10.3389/fphar.2022.1095734 (PMC9870248; doi:10.3389/fphar.2022.1095734)
Supplement: Supplementary file 1 [file DataSheet1.DOCX]

**Supplementary material**

**Supplementary Tables**

**TABLE S1 | PubMed Retrieval strategy.**

| No | Query |
| --- | --- |
| #1 | "Nasopharyngeal Carcinoma"[Mesh] |
| #2 | (((((Nasopharyngeal Carcinoma[Title/Abstract]) OR (Carcinoma, Nasopharyngeal[Title/Abstract])) OR (Carcinomas, Nasopharyngeal[Title/Abstract])) OR (Nasopharyngeal Carcinomas[Title/Abstract])) OR (nasopharyngeal cancer[Title/Abstract])) OR (NPC[Title/Abstract]) |
| #3 | #1 OR #2 |
| #4 | "Immune Checkpoint Inhibitors"[Mesh] |
| #5 | ((((((((((((((((((((((((((((Immune Checkpoint Inhibitors[Title/Abstract]) OR (Checkpoint Inhibitors, Immune[Title/Abstract])) OR (Immune Checkpoint Inhibitor[Title/Abstract])) OR (Checkpoint Inhibitor, Immune[Title/Abstract])) OR (Immune Checkpoint Blockers[Title/Abstract])) OR (Checkpoint Blockers, Immune[Title/Abstract])) OR (Immune Checkpoint Blockade[Title/Abstract])) OR (Checkpoint Blockade, Immune[Title/Abstract])) OR (Immune Checkpoint Inhibition[Title/Abstract])) OR (Checkpoint Inhibition, Immune[Title/Abstract])) OR (PD-1 Inhibitors[Title/Abstract])) OR (PD 1 Inhibitors[Title/Abstract])) OR (PD-1 Inhibitor[Title/Abstract])) OR (Inhibitor, PD-1[Title/Abstract])) OR (PD-1 antibody[Title/Abstract])) OR (PD-L1 antibody[Title/Abstract])) OR (PD 1 Inhibitor[Title/Abstract])) OR (Programmed Cell Death Protein 1 Inhibitor[Title/Abstract])) OR (Programmed Cell Death Protein 1 Inhibitors[Title/Abstract])) OR (PD-L1 Inhibitors[Title/Abstract])) OR (PD L1 Inhibitors[Title/Abstract])) OR (PD-L1 Inhibitor[Title/Abstract])) OR (PD L1 Inhibitor[Title/Abstract])) OR (PD-L1 antibody[Title/Abstract])) OR (Programmed Death-Ligand 1 Inhibitors[Title/Abstract])) OR (Programmed Death Ligand 1 Inhibitors[Title/Abstract])) OR (PD-1-PD-L1 Blockade[Title/Abstract])) OR (Blockade, PD-1-PD-L1[Title/Abstract])) OR (PD 1 PD L1 Blockade[Title/Abstract]) |
| #6 | #4 OR #5 |
| #7 | #3 AND #6 |

**TABLE S2 |** Embase Retrieval strategy.

| No | Query |
| --- | --- |
| #40 | #10 AND #39 |
| #39 | #11 OR #12 OR #13 OR #14 OR #15 OR #16 OR #17 OR #18 OR #19 OR #20 OR #21 OR #22 OR #23 OR #24 OR #25 OR #26 OR #27 OR #28 OR #29 OR #30 OR #31 OR #32 OR #33 OR #34 OR #35 OR #36 OR #37 OR #38 |
| #38 | 'pd 1 pd l1 blockade':ab,ti |
| #37 | 'blockade, pd-1-pd-l1':ab,ti |
| #36 | 'pd-1-pd-l1 blockade':ab,ti |
| #35 | 'programmed death ligand 1 inhibitors':ab,ti |
| #34 | 'programmed death-ligand 1 inhibitors':ab,ti |
| #33 | 'pd-l1 antibody':ab,ti |
| #32 | 'pd-l1 inhibitor':ab,ti |
| #31 | 'pd l1 inhibitors':ab,ti |
| #30 | 'pd-l1 inhibitors':ab,ti |
| #29 | 'programmed cell death protein 1 inhibitors':ab,ti |
| #28 | 'programmed cell death protein 1 inhibitor':ab,ti |
| #27 | 'pd 1 inhibitor':ab,ti |
| #26 | 'pd-1 antibody':ab,ti |
| #25 | 'inhibitor, pd-1':ab,ti |
| #24 | 'pd-1 inhibitor':ab,ti |
| #23 | 'pd 1 inhibitors':ab,ti |
| #22 | 'pd-1 inhibitors':ab,ti |
| #21 | 'checkpoint inhibition, immune':ab,ti |
| #20 | 'immune checkpoint inhibition':ab,ti |
| #19 | 'checkpoint blockade, immune':ab,ti |
| #18 | 'immune checkpoint blockade':ab,ti |
| #17 | 'checkpoint blockers, immune':ab,ti |
| #16 | 'immune checkpoint blockers':ab,ti |
| #15 | 'checkpoint inhibitor, immune':ab,ti |
| #14 | 'immune checkpoint inhibitor':ab,ti |
| #13 | 'checkpoint inhibitors, immune':ab,ti |
| #12 | 'immune checkpoint inhibitors':ab,ti |
| #11 | 'immune checkpoint inhibitor'/exp |
| #10 | #1 OR #2 OR #3 OR #4 OR #5 OR #6 OR #7 OR #8 OR #9 |
| #9 | 'npc':ab,ti |
| #8 | 'nasopharyngeal cancer':ab,ti |
| #7 | 'nasopharyngeal carcinomas':ab,ti |
| #6 | 'carcinomas, nasopharyngeal':ab,ti |
| #5 | 'carcinoma, nasopharyngeal':ab,ti |
| #4 | 'nasopharynx cancer':ab,ti |
| #3 | 'nasopharyngeal carcinoma':ab,ti |
| #2 | 'nasopharynx carcinoma':ab,ti |
| #1 | 'nasopharynx carcinoma'/exp |

**TABLE S3** | Newcastle-Ottawa Scale (NOS) for Single arm studies.

| Newcastle-Ottawa Scale (NOS) for Single arm studies | | | | |
| --- | --- | --- | --- | --- |
| Study | Selection | Comparability | Exposure | Total score |
| P Economopoulou | ******* | ****** | ****** | 7 |
| WF Fang | ****** | ****** | ****** | 6 |
| C Hus | ******* | ***** | ****** | 5 |
| BBY Ma | ******* | ****** | ******* | 8 |
| YX Ma | ******* | ****** | ****** | 7 |
| H Sato | ****** | ****** | ******* | 7 |
| FH Wang | ******* | ****** | ******* | 8 |

*: one score

**TABLE S4 |** Publish biased assessments.

| Outcome | P value for Egger test | P value for Begg test |
| --- | --- | --- |
| Single-armed experiment | | |
| ORR | 0.244 | 0.180 |
| OS | 0.196 | 0.583 |
| PFS | 0.074 | 0.095 |
| Randomized controlled trial | | |
| ORR | 0.294 | 0.117 |
| OS | 0.718 | 1.000 |
| PFS | 0.528 | 1.040 |

Abbreviation: OS: Overall survival; PFS: progression free survival; ORR: Objective response rate

**TABLE S5|** Results of meta-analysis of adverse events.

| adverse event | Any grade | | | | | Grade≥3 | | | | |
| --- | --- | --- | --- | --- | --- | --- | --- | --- | --- | --- |
|  | study | Heterogeneity | | ES (95%CI) | P | study | Heterogeneity | | ES (95%CI) | P |
|  |  | P | I^2^(%) |  |  |  | P | I^2^(%) |  |  |
| Rash | 7 | 0 | 87.2 | 0.18(0.09,0.27) | 0.000 | 2 | 0.29 | 9.9 | 0.02(0,0.04) | 0.014 |
| Leukopenia | 3 | 0 | 99.6 | 0.66(0.23,1.10) | 0.003 | 2 | 0.465 | 0 | 0.64(0.58,0.70) | 0.000 |
| Anemia | 7 | 0 | 99.8 | 0.36(-0.06,0.78) | 0.095 | 5 | 0 | 97.8 | 0.18(0.05,0.31) | 0.008 |
| Neutropenia | 3 | 0 | 99.6 | 0.63(0.13,1.12) | 0.013 | 4 | 0 | 99.3 | 0.31(0.05,0.57) | 0.020 |
| Vomiting | 2 | 0.054 | 73.1 | 0.62(0.51,0.73) | 0.000 | 2 | 0.058 | 72.1 | 0.04(0.00,0.09) | 0.080 |
| Thrombocytopenia | 3 | 0 | 99.7 | 0.48(-0.09,1.05) | 0.096 | 3 | 0 | 98.5 | 0.24(-0.03,0.52) | 0.080 |
| Decreased appetite | 3 | 0 | 98.7 | 0.40(0.04,0.77) | 0.03 | 2 | 0.701 | 0 | 0.01(0.00,0.02) | 0.171 |
| Constipation | 4 | 0 | 98.6 | 0.28(-0.02,0.59) | 0.070 | NR | | | | |

Abbreviation: CI: confidence interval; NR: Not reported

**Supplementary Figures**


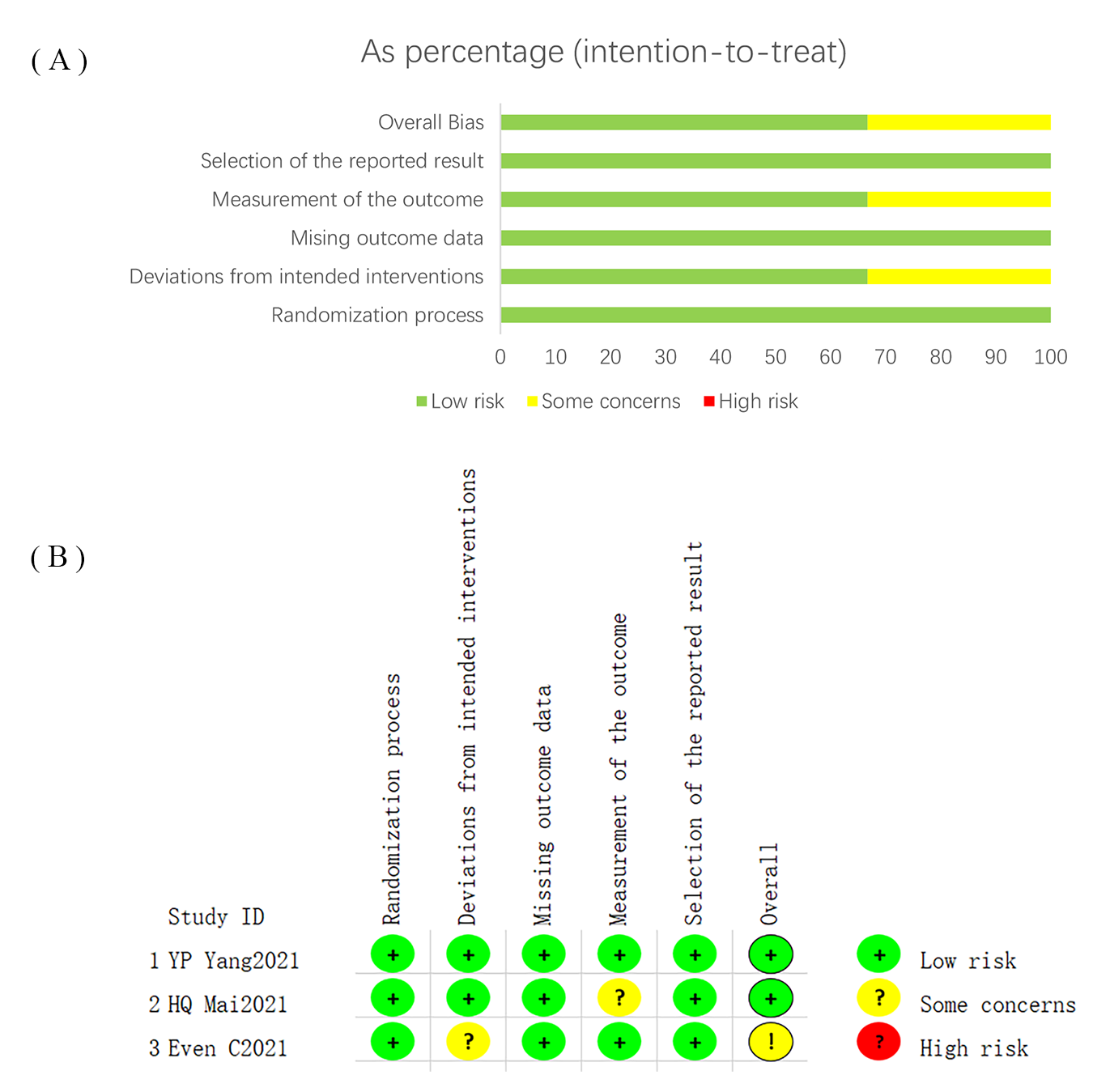


**FIGURE S1 |** Risk of bias assessment. (A) Risk of bias graph; (B) Risk of bias summary.


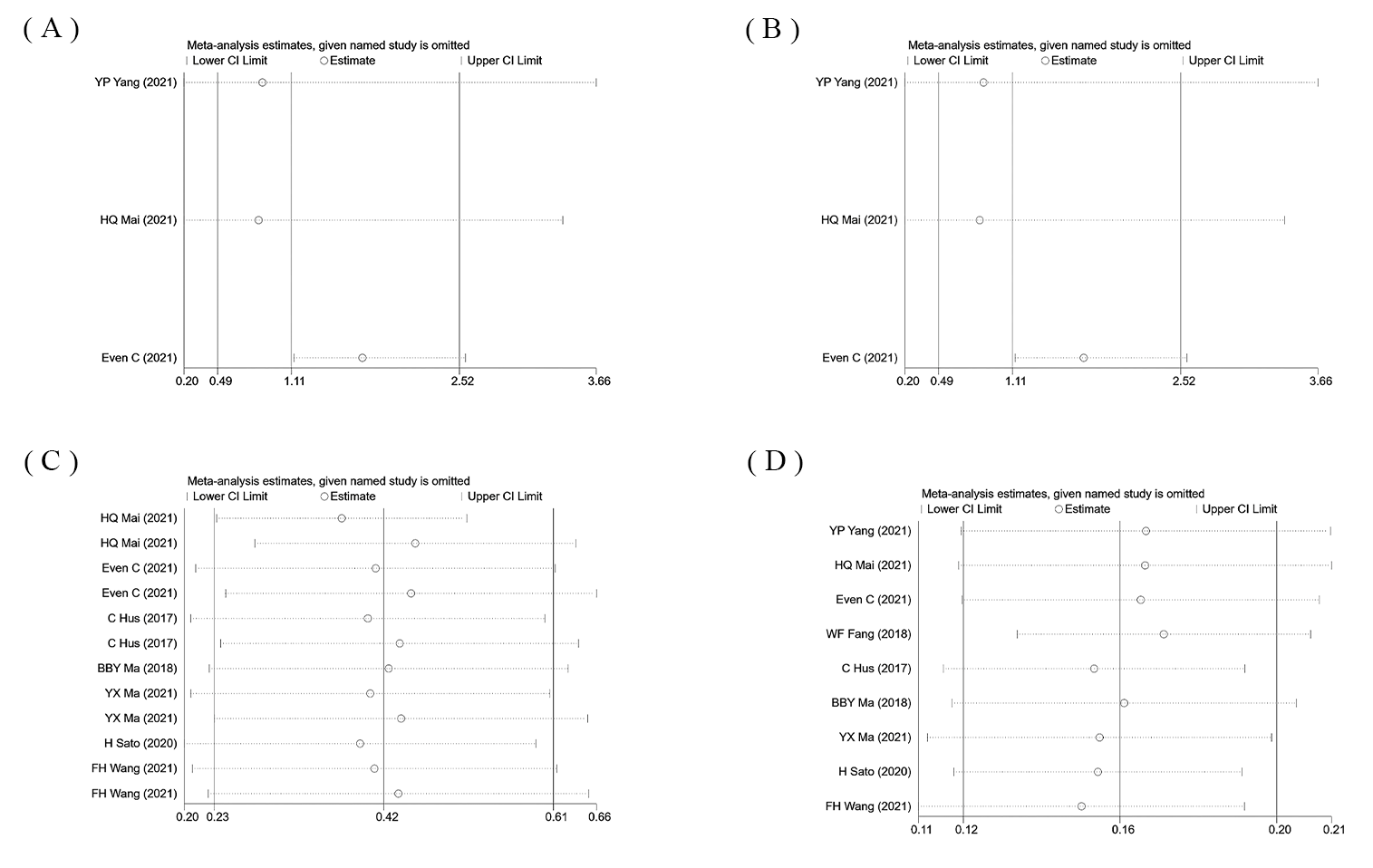


**FIGURE S2 |** Sensitivity analysis. (A) ORR Sensitivity analysis- RCT; (B) ORR Sensitivity analysis- single arm study; (C) OS Sensitivity analysis- single arm study; (D) PFS Sensitivity analysis- single arm study.
